# Editorial: Mouse Models of Hematopoietic Stem Cell Transplantation

**DOI:** 10.3389/fimmu.2022.882592

**Published:** 2022-04-06

**Authors:** Antiopi Varelias, Ping Zhang, Daigo Hashimoto

**Affiliations:** ^1^ Cancer Research Program, QIMR Berghofer Medical Research Institute, Brisbane, QLD, Australia; ^2^ Faculty of Medicine, University of Queensland, St Lucia, QLD, Australia; ^3^ School of Biomedical Sciences, Queensland University of Technology, Brisbane, QLD, Australia; ^4^ Clinical Research Division, Fred Hutchinson Cancer Research Center, Seattle, WA, United States; ^5^ Department of Hematology, Hokkaido University Faculty of Medicine, Sapporo, Japan

**Keywords:** hematopoietic stem cell transplantation, graft-versus-host disease, mouse models, regulatory T cells, antigen presentation, microbiota, opportunistic infections

Bone marrow or hematopoietic stem cell transplantation (HSCT) is a curative treatment option for hematological malignancies, several non-malignant hematological disorders and severe combined immune deficiencies. However, relapse, graft-versus-host disease (GVHD) and opportunistic infections represent major complications following allogeneic HSCT which compromise the survival and quality-of-life of the recipient, limiting the wider application of this immunotherapy. Basic research has significantly advanced our understanding of the underlying mechanisms of these complications and has led to the development of promising pharmaceutical, immune and cellular therapies. Despite intensive research and development, GVHD, infection and relapse remain significant clinical problems and represent major unmet needs in HSCT.

This *Frontiers In Immunology* Research Topic presents the latest insights from preclinical studies of HSCT, highlighting opportunities and challenges, and sheds light on the future of HSCT. The collection is comprised predominantly of review articles which focus on GVHD pathophysiology, regulatory T cell-mediated immune tolerance, antigen presentation, microbiota/metabolite modulation of GVHD, intestinal immunopathology and infectious pulmonary complications, with an emphasis on the biological relevance to the clinic and translational potential.

The perspective article by Teshima and Hill provides a historical overview of major experimental concepts in bone marrow transplantation discovered using murine models and the translation of these findings into clinical practice, highlighting the pivotal insights afforded by murine models.

Since the discovery of regulatory T cells (Tregs) by Sakaguchi in 1995, Tregs have been of great interest to researchers studying immune tolerance and regulation after HSCT. In this collection, Guo et al. review the biology of Tregs, their role in GVHD and GVL and the challenges related to Treg adoptive cell therapy to treat and/or prevent GVHD. The authors review Treg expansion strategies and discuss new sources of Tregs to address challenges encountered with this therapy. In the review by Ikegawa and Matsuoka, the authors detail the importance of Treg homeostasis post-HSCT and discuss strategies to manipulate this to modulate post-HSCT immunity to treat and/or prevent GVHD.

The cardinal feature of GVHD is immune-mediated tissue pathology. Gastrointestinal GVHD is the most challenging to treat and the greatest cause of GVHD-related mortality. In this collection, Ara and Hashimoto review historical and recent advances in our understanding of the cellular and molecular mechanisms of GVHD-induced tissue damage in the gastrointestinal tract, providing important novel insights garnered from mouse models which inform translational potential. With technological advances in genomic/metagenomic sequencing and metabolomics, the ability to study intestinal microorganisms and their secreted molecules in HSCT has rapidly generated new knowledge in the field. In this collection, Fujiwara provides an in-depth review of the intestinal microbiota, metabolite and epithelial cell crosstalk critical in GVHD regulation, highlighting the complex relationships and potential microbiota-based therapeutic strategies.

Conditioning-induced toxicity, immunosuppressive therapies and alloimmunity permit opportunistic infections post-HSCT, a major cause of morbidity and mortality. Zhou and Moore review murine models of infectious pulmonary complications after HSCT (e.g. bacterial, fungal and viral) focusing on host-pathogen interactions and the important insights garnered from these studies.

The therapeutic effect of HSCT, mediated by alloimmunity towards malignant cells (graft-versus-leukemia/tumor; GVL), is compromised by detrimental alloimmunity towards recipient tissues (GVHD). Antigen presentation is central to these two processes, however the ability to delineate between these remains elusive and is considered the holy grail in HSCT. Koyama and Hill review the field and discuss recent advances uncovered by the use of antigen-specific murine models of GVHD, providing novel insights for the translational potential of targeting this pathway. Critically, the authors review antigen presentation in xenograft transplantation models used to study GVHD and GVL and discuss the controversies associated with the interpretation of findings from these models.

Chronic GVHD continues to be a major clinical problem in HSCT survivors. Song et al. review important mechanistic findings identified using murine models of chronic GVHD which reflect features characteristic of the disease in humans. The authors highlight low P-selectin glycoprotein ligand 1 expressing CD4 T cells as key players in cGVHD pathophysiology. Original research presented by Choi et al. revealed that deletion of the endoplasmic reticulum stress protein, XBP-1, reduced B cell activity and the ability to stimulate allogeneic CD4 T cells *via* a regulated IRE-1a-dependent decay pathway which led to a reduction in cGVHD. These preclinical findings demonstrate targeting XBP-1 a potentially useful strategy to ameliorate cGVHD.

Finally, Shaikh et al. present a detailed description of the development of a murine model of orthotopic mesenteric lymph node transplantation to study immune cell responses and migration in the gastrointestinal tract. The authors determined its utility in a HSCT setting and suggest potential for broader application.

In summary, murine models have proven to be instrumental for advancement of our understanding of the complications that occur following HSCT. As demonstrated in this collection, murine models of HSCT have led to the discovery of new scientific knowledge, permitted established concepts to be challenged and emerging concepts to be explored, enabled the development of new therapeutics and provided important insights that have guided clinical studies. With the development of new ideas, technologies and tools, murine models of HSCT will continue to empower academic research and translation into the clinic in the future.

## Author Contributions

All authors contributed to the article and approved the submitted version.

## Conflict of Interest

The authors declare that the research was conducted in the absence of any commercial or financial relationships that could be construed as a potential conflict of interest.

## Publisher’s Note

All claims expressed in this article are solely those of the authors and do not necessarily represent those of their affiliated organizations, or those of the publisher, the editors and the reviewers. Any product that may be evaluated in this article, or claim that may be made by its manufacturer, is not guaranteed or endorsed by the publisher.

